# Supplementary figures and images for: Non-canonical start codons confer context-dependent advantages in carbohydrate utilization for commensal E. coli in the murine gut
Source: Nat Microbiol. 2024 Aug 19;9(10):2696–709. doi: 10.1038/s41564-024-01775-x (PMC11445065; doi:10.1038/s41564-024-01775-x)

**Supplementary data 1: Uncropped western blot used in Figures 2A and 4A.**

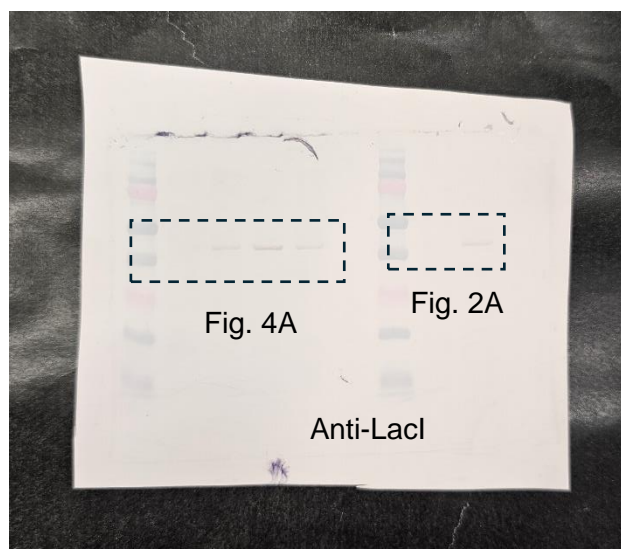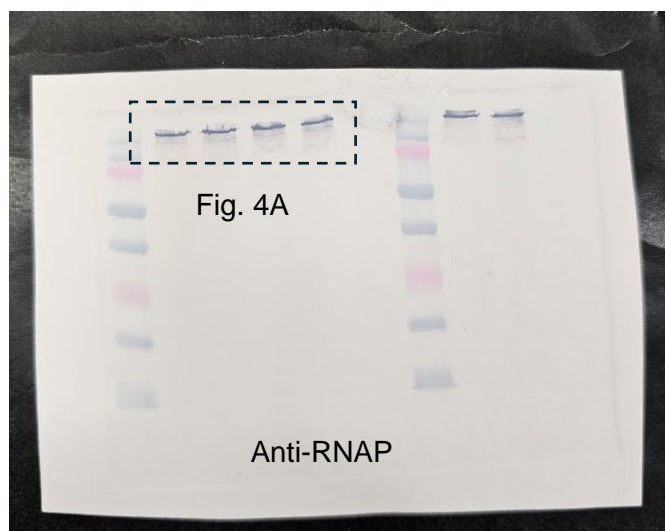

Supplement: Supplementary file 12 — Unprocessed gels. [file 41564_2024_1775_MOESM12_ESM.pdf]
